# Diabetes Mellitus and Cardiovascular Disease: Exploring Epidemiology, Pathophysiology, and Treatment Strategies

**DOI:** 10.31083/j.rcm2512436

**Published:** 2024-12-11

**Authors:** Nawfal Hasan Siam, Nayla Nuren Snigdha, Noushin Tabasumma, Irin Parvin

**Affiliations:** ^1^Department of Pharmacy, School of Pharmacy and Public Health, Independent University, Bangladesh (IUB), 1229 Dhaka, Bangladesh; ^2^Department of Biomedical Science, School of Health and Life Sciences, Teesside University, TS1 3BX Middlesbrough, UK

**Keywords:** diabetes mellitus, insulin resistance, cardiovascular and obesity, hypertension, hyperglycemia, cardiovascular drugs

## Abstract

Diabetes mellitus (DM) affects 537 million people as of 2021, and is projected to rise to 783 million by 2045. This positions DM as the ninth leading cause of death globally. Among DM patients, cardiovascular disease (CVD) is the primary cause of morbidity and mortality. Notably, the prevalence rates of CVD is alarmingly high among diabetic individuals, particularly in North America and the Caribbean (46.0%), and Southeast Asia (42.5%). The predominant form of CVD among diabetic patients is coronary artery disease (CAD), accounting for 29.4% of cases. The pathophysiology of DM is complex, involving insulin resistance, β-cell dysfunction, and associated cardiovascular complications including diabetic cardiomyopathy (DCM) and cardiovascular autonomic neuropathy (CAN). These conditions exacerbate CVD risks underscoring the importance of managing key risk factors including hypertension, dyslipidemia, obesity, and genetic predisposition. Understanding the genetic networks and molecular processes that link diabetes and cardiovascular disease can lead to new diagnostics and therapeutic interventions. Imeglimin, a novel mitochondrial bioenergetic enhancer, represents a promising medication for diabetes with the potential to address both insulin resistance and secretion difficulties. Effective diabetes management through oral hypoglycemic agents (OHAs) can protect the cardiovascular system. Additionally, certain antihypertensive medications can significantly reduce the risk of diabetes-related CVD. Additionally, lifestyle changes, including diet and exercise are vital in managing diabesity and reducing CVD risks. These interventions, along with emerging therapeutic agents and ongoing clinical trials, offer hope for improved patient outcomes and long-term DM remission. This study highlights the urgent need for management strategies to address the overlapping epidemics of DM and CVD. By elucidating the underlying mechanisms and risk factors, this study aims to guide future perspectives and enhance understanding of the pathogenesis of CVD complications in patients with DM, thereby guiding more effective treatment strategies.

## 1. Introduction

Diabetes mellitus (DM) is a growing global health concern, currently regarded as an epidemic [1]. As of 2021, an estimated 537 million individuals aged 20 to 79 were living with DM; projections indicate this number will rise to 643 million by 2030 and 783 million by 2045, posing a growing challenge for patients and healthcare professionals [2]. According to the World Health Organization (WHO), DM is currently the ninth leading cause of death worldwide [3], with 1.5 million deaths in 2019 directly attributed to the disease [4, 5]. DM is a chronic condition that increases the risk of developing cardiovascular disease (CVD) and related complications [6]. CVD is a leading cause of death globally and is the primary cause of mortality among in individuals with DM [4]. In developed countries, heart failure is notably the most common cause of death [7]. There is a strong correlation between DM and CVD, with a study showing that 44% of deaths among individuals with type 1 DM (T1DM) and 52% among those with type 2 DM (T2DM) are due to CVD [8]. The risk of CVD increases proportionally with mounting blood sugar levels, even before blood glucose reaches diabetic thresholds [9, 10]. Given this strong association, one of the major goals of DM treatment is the early identification and management of potential CVD risks [9]. Diabetic adults are 2–4 times more likely to die from myocardial infarction (MI), ischemic heart disease, congestive heart failure (CHF), and stroke compared to non-diabetic individuals [1]. Factors such as hyperglycemia, hypertension, obesity, dyslipidemia, and insulin resistance (IR) contribute to the development of CVD in diabetic patients [6, 11].

The progression of CVD in DM has been linked to heightened oxidative stress, hypercoagulability, endothelial dysfunction, and autonomic neuropathy [12]. Moreover, CVD in DM is also linked to abnormalities in genetic, epigenetic, and cellular signaling metabolic pathways, often triggered by factors including glucose toxicity, advanced glycation end-products (AGEs), and smoking [13, 14]. Atherosclerotic cardiovascular disease (ASCVD), a major type of CVD, develops through plaque formation driven by IR and high blood sugar levels. In diabetic, pre-diabetic, and obese individuals, IR accelerates atherosclerosis by promoting vascular inflammation, diabetic dyslipidemia, vascular stiffness, and hypertension [6]. Given the rapid progression and significant economic impact of DM, effective management is imperative. Notably, CVD remains the leading cause of mortality among diabetic patients, underscoring the critical need for risk reduction strategies [15].

Many complex DM dependent metabolic processes raise the risk of CVD, underscoring the importance of therapeutic strategies that effectively reduce CVD complications [6, 12]. Historically, improving glycemic control has been the primary approach to reducing the risk of CVD, MI, and CVD-related mortality in diabetic individuals, as supported by the Swedish National Diabetes Registry [16, 17]. In terms of medication, cardio-renal protective agents like sodium-glucose co-transporter-2 (SGLT-2) inhibitors have shown benefits in reducing renal complications and multiple CVD risks in individuals with T2DM [18]. Glucagon-like peptide-1 (GLP-1) receptor agonists have also demonstrated cardiovascular benefits in diabetic patients [18, 19]. Tirzepatide which acts as a dual agonist on both gastric inhibitory polypeptide (GIP) and GLP-1 receptors has shown significant improvement in glycemic control in patients with T2DM, while also lowering low-density lipoprotein (LDL) levels and improving blood pressure (BP) [20]. For diabetic individuals at risk of developing atherosclerosis and dyslipidemia, lipid lowering drugs like statins have improved cardiovascular function. In such patients, if statins are ineffective, medicines like Ezetimibe and proprotein convertase subtilisin/kexin type 9 (PCSK9) inhibitors can be used to lower low-density lipoprotein-cholesterol (LDL-C) levels [21].

Clearly, CVD is the leading cause of mortality and morbidity in diabetic patients [6]. The aim of this study is to comprehensively investigate the interplay between DM and CVD by exploring epidemiological trends and underlying pathophysiological mechanisms. Additionally, the study seeks to identify and evaluate effective management strategies to mitigate the combined impact of DM and CVD. Ultimately, this could enhance clinical outcomes and improve quality of life for those affected.

## 2. Materials and Method

The information for this article was gathered through electronic searches using various international scientific databases, such as PubMed, Cochrane library and Google Scholar. Specific keywords such as ‘Diabetes Mellitus’, ‘Insulin resistance’, ‘CVD’, ‘Obesity’, ‘Hypertension’, ‘Hyperglycemia’, ‘Cardiovascular and Diabetes medications’ were utilized. The search covered a wide timeframe without restrictions. Of the studies reviewed, approximately 0.5% of the data retrieved predating 2000, 27.5% were published between 2000 and 2012, with the remaining 72% dating from the last decade. Initially, about 400 papers were reviewed, following a primary screening process, nearly 244 papers were selected for critical examination and summarization for the current review. Additionally, the reference list was manually checked to eliminate any duplicates, ensuring the uniqueness and relevance of each included study.

## 3. Epidemiology

DM is a progressive and chronic condition that occurs due to increased glucose levels in the body. Prior to the development of systemic population-based studies, there were difficulties in determining DM prevalence both in the United States and globally [22]. It is a chronic systemic condition impacting multiple organ systems leading to complications that significantly affect mortality and rates. Hence, the DM epidemic contributes to a variety of complex abnormalities converging on the cardiovascular system [23].

T1DM patients face a notably higher cumulative mortality rate (CMR) from coronary artery disease (CAD) compared to non-diabetics. By the age of 55, their total CMR for CAD was 35 ± 5%, compared to only 8% of male and 4% of female non-diabetic participants in the Framingham Heart Study [24]. For T1DM patients aged 45–59, the CMR was 33% for clinical coronary heart disease (CHD), including angina and nonfatal MI, as well as asymptomatic CAD identified through stress tests [24]. The Pittsburgh Epidemiology of Diabetes Complications (EDC) study highlighted CAD events as the primary cause of death among T1DM patients [24]. The majority of CAD events occur at a rate of 0.98% per year between the ages of 28 and 38, increasing to over 3% per year by age 55 [24]. Additionally, the standardized mortality ratios (SMR) for CVD were reported as 8.8 for males and 24.7 for females in the Allegheny County Type 1 Diabetes Registry [24].

The EURODIAB Insulin dependent DM (IDDM) complications study, involving 3250 T1DM patients across 16 European countries, reported a CVD prevalence of 9% in males and 10% in females [24]. Thes rates increased with age and DM duration from 6% in those aged 15–29 to 25% in those aged 45–59 [24]. According to the 2020 Diabetes Fact Sheet for Korea, DM prevalence among adults aged 30 years or older was 13.8%, corresponding to 4.94 million individuals . Of these, 61.3% had hypertension, a known risk factor for both CVD and heart failure. The coexistence of these conditions significantly elevates the risk of developing CVD [25, 26]. Data from the Korean Acute Heart Failure Registry (KorAHF) from 2004 to 2009 revealed that 36% of heart failure patients also had a diagnosis of DM [25, 27].

**Fig. 1.**
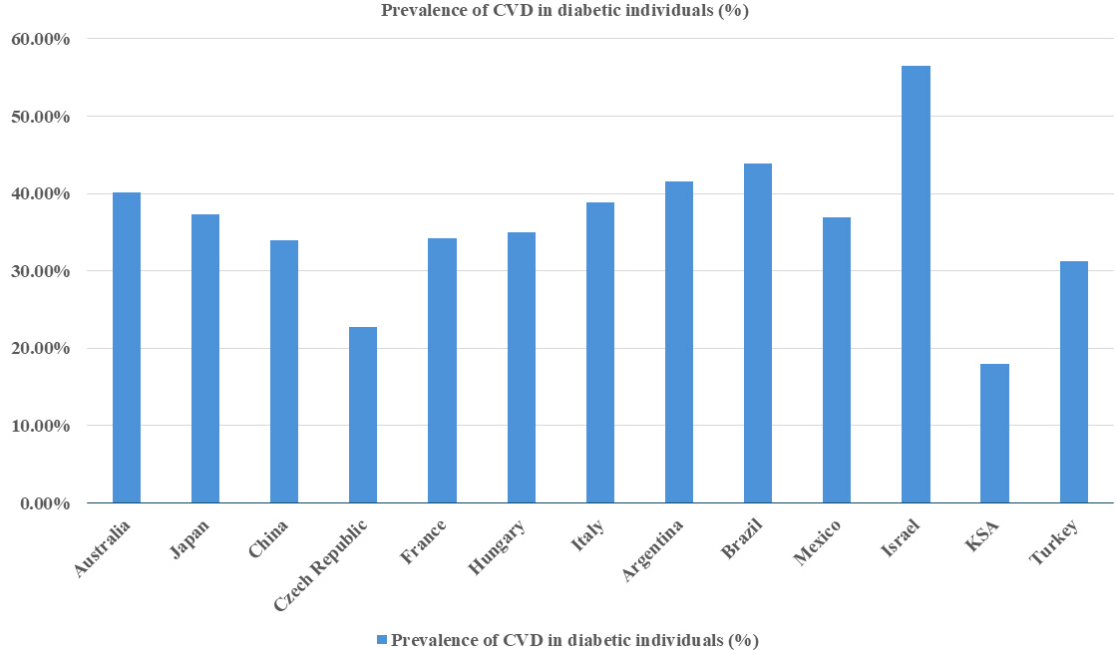
**Global prevalence of cardiovascular disease among diabetic individuals**. This figure illustrates the distribution of CVD in individuals diagnosed with DM in 13 countries. This visualization aims to underscore the global impact of cardiovascular complications among those with DM. CVD, cardiovascular disease; DM, diabetes mellitus; KSA, Kingdom of Saudi Arabia.

A comprehensive study involving 9823 individuals from 13 countries investigated global variations the prevalence of various types of CVD in diabetic individuals, including cerebrovascular disease, CHD, heart failure, cardiac arrhythmia or conduction abnormalities, aortic disease, and peripheral artery disease (PAD) [28]. The prevalence rates varied significantly by country: Australia (40.1%), China (33.9%), Japan (37.3%), Czech Republic (22.8%), France (34.2%), Hungary (35.0%), Italy (38.8%), Argentina (41.5%), Brazil (43.9%), Mexico (36.9%), Israel (56.5%), KSA (18.0%), Turkey (31.2%). Among the various forms of CVD analyzed, the majority of cases (85.8%, n = 3074) were atherosclerotic, with the weighted ASCVD estimated at 31.8%. Furthermore, a significant portion of the participants (80.4%) had hypertension, underscoring the frequent coexistence of these risk factors in diabetic populations (Fig. 1) [28].

**Fig. 2.**
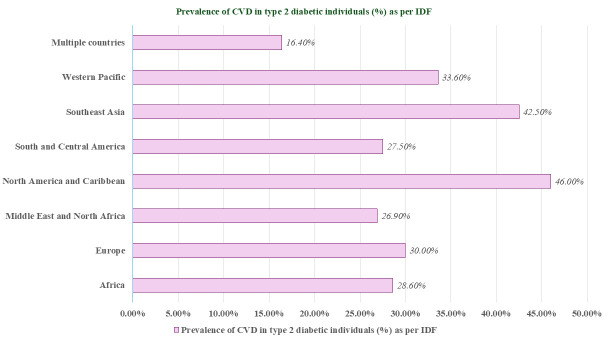
**Geographic variation in CVD prevalence rates among T2DM individuals**. This figure illustrates the prevalence rates of CVD among individuals with T2DM across various global regions, according to the International Diabetes Federation’s (IDF) classification system. The chart details prevalence percentages in regions such as Africa, Europe, the Middle East and North Africa, North America and the Caribbean, South and Central America, Southeast Asia, the Western Pacific (including China), and other nations. Notably, the highest CVD prevalence occurs in North America and the Caribbean, Southeast Asia, and the Western Pacific. CVD, cardiovascular disease; T2DM, type 2 diabetes mellitus.

The International Diabetes Federation’s (IDF) classification system [29] reveals global variations in the prevalence of CVD in type 2 diabetic (T2D) patients. Geographically, the prevalence rates are as follows: Africa (28.6%), Europe (30.0%), the Middle East and North Africa (26.9%), North America and the Caribbean (46.0%), South and Central America (27.5%), Southeast Asia (42.5%), the Western Pacific (which includes China) (33.6%), and numerous nations (16.4%) (Fig. 2). A visual depiction of the prevalence rates of CVD among individuals with T2DM, categorized by region, is presented in Fig. 2. The regions with the greatest CVD prevalence were North America and Caribbean (46.0%; N = 4,327,503), Southeast Asia (42.5%, N = 537), and the Western Pacific (including China) (33.6%; N = 44,062). The majority of CVD cases were identified as CAD (29.4%) [29, 30].

The REasons for Geographic and Racial Differences in Stroke (REGARDS) highlighted disparities in cardiovascular health (CVH), particularly between African-American and other participants. Poorer baseline CVH metrics were found among African-Americans, contributing to a smaller reduction in DM risk compared to other participants. Although good blood pressure and body mass index (BMI) were linked to a lower risk of DM in the rest of the participants, this effect was not as strong in the African-American population. Physical activity and diet also reduced DM risk in rest of the participants but were less effective in African-Americans. This disparity may stem from co-existing factors like adiposity, inflammation, endothelial dysfunction and socioeconomic status. The high prevalence of hypertension and DM among African-Americans, as observed in the REGARDS study along with the data from IDF, underscores the need for further research. Investigating the mechanistic links underlying pathophysiological differences is crucial to better understand and address these disparities in health outcomes [31].

A nationwide prospective study conducted over seven years involved 500,000 individuals aged 30 to 79 across ten regions in China. The study revealed that individuals with DM faced approximately twice the risk of all-cause mortality compared to those without DM [32, 33]. In a separate 2018 study conducted in China, regional variations were observed in the rates of CHD and stroke among T2DM patients. The study identified several factors contributing to these differences, including lifestyle factors such as diet and physical activity, cold ambient temperatures, high dietary salt intake (particularly in the Northeast and North), and potential genetic differences [34].

The prevalence of ASCVD in T2DM in the U.S. was found to be 71% in individuals aged more than 65 years [35]. In the similar Chennai Urban Population Study (CUPS), conducted in both diabetics and non-diabetics, the occurrence of CAD among diabetic subjects was found to be 21.4% (previously diagnosed DM, 25.3%, and newly diagnosed, 13.1%), which was significantly greater than the rate of 14.9% amongst subjects with impaired glucose tolerance (IGT) and 9.1% amongst subjects with normal glucose tolerance (NGT). Overall, the prevalence of known MI was three times greater in diabetic individuals. However, the CUPS study also showed that the prevalent risk for CAD escalated even in the IGT stage [36, 37, 38].

A large-scale population-based cohort study was carried out, involving nearly 40,000 T2DM patients from the Swedish National Diabetes Register. The patients were closely monitored over a period of roughly six years. The trial results during follow-up showed a 21% reduction in overall mortality among T2DM patients (hazard ratio [HR] = 0.79; 95% confidence interval [CI]: 0.78–0.80). In contrast, non-diabetic controls matched for age, sex, and nationality, exhibited a 31% decrease in mortality (HR = 0.69; 95% CI: 0.68–0.70). Conversely, the overall death rate among participants with T1DM was 13% higher among controls compared to T2DM patients within the same registry [33, 39]. These disparities may be attributed to differences in healthcare availability, lifestyle choices, socioeconomic factors, and genetic predispositions. Numerous clinical and epidemiological cohort studies have been undertaken worldwide on individuals with DM. These studies focused on thousands of participants with either T1DM or T2DM in diverse global regions and healthcare settings. These studies revealed that diabetic patients have a risk of CVD, complications, and mortality approximately 1.5 to 6 times higher compared to non-diabetic individuals [40]. The elevated risk seems to affect people regardless of DM type. However, the magnitude of the threat varies depending on factors such as the type of DM, presence of CVD risk factors and comorbidities, severity and duration of hypoglycemia relative to the CVD event, follow-up period, and the extent of adjustment for potent confounding factors [40]. This highlights the growing body of evidence implicating hypoglycemia in the deterioration of cardiovascular health, even if other factors may contribute to the underlying pathology. Hence, further research is essential to improve the understanding of the connection between hypoglycemia and its contribution to cardiovascular events [41].

## 4. Pathophysiology of Events

### 4.1 Diabetes Mellitus (DM)

**Fig. 3.**
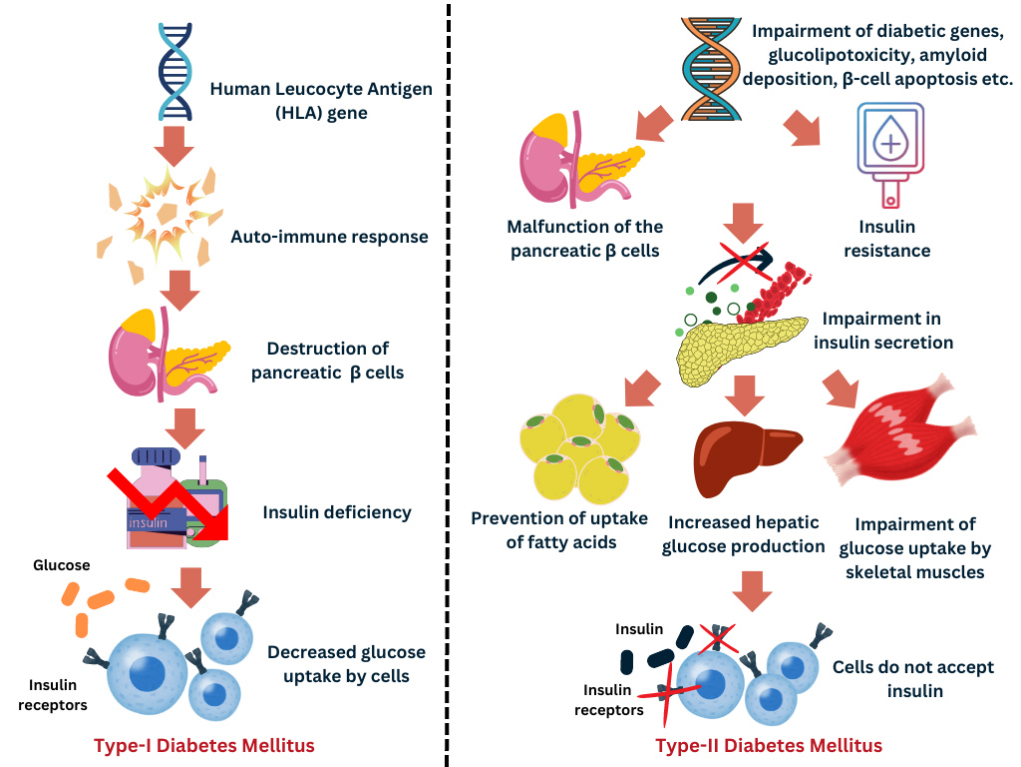
**Pathophysiology of type 1 diabetes mellitus (T1DM) and type 2 diabetes mellitus (T2DM)**. This figure illustrates the distinct pathophysiological mechanisms underlying the development of T1DM and T2DM. Autoimmune processes in T1DM lead to the elimination of pancreatic β-cells that are responsible for insulin production. This process involves CD4+ and CD8+ T cells, as well as the infiltration of macrophages which damage the pancreatic islets, leading to inadequate secretion of insulin. In T2DM, pancreatic β-cell dysfunction impairs insulin secretion while IR hinders insulin action. These processes collectively contribute to dysfunction in the maintenance of glucose homeostasis and the onset of metabolic disorders associated with each type of DM. DM, diabetes mellitus.

DM is a chronic metabolic disorder characterized by hyperglycemia; a pathological condition defined by substantially elevated blood sugar levels. Hyperglycemia is caused by defects in either insulin production, insulin action, or a combination of the two, resulting in long-term and complex dysfunctions of carbohydrate, protein, and fat metabolism [42]. The long-term consequences related to DM include complications such as diabetic retinopathy (which carries a potential risk of blindness), nephropathy (resulting in kidney failure), neuropathy (leading to foot ulcers, Charcot’s joints, and amputations), autonomic neuropathy (leading to gastrointestinal disorders), sexual dysfunction, and cardiovascular abnormalities. Individuals diagnosed with DM often experience hypertension and impaired lipoprotein metabolism. As a consequence, they are more susceptible to peripheral artery, cerebrovascular, cardiovascular, and atherosclerotic disorders [43]. On the basis of its etiology and pathogenesis, DM can be classified into two major types: T1DM and T2DM. T1DM involves the autoimmune destruction of the insulin-producing cells in the pancreas. Specifically, the immune system’s CD4+ and CD8+ T cells target and destroy these cells, while macrophages infiltrate and damage the pancreatic islets. Insufficient insulin secretion results from the autoimmune destruction of pancreatic β-cells, leading to various metabolic disorders associated with T1DM [44]. In T2DM, the mechanisms that maintain normal physiological glucose levels break down, leading to two major pathological defects: impaired insulin secretion due to pancreatic β-cell dysfunction and impaired insulin action due to IR [44, 45]. It is evident that the presence of DM escalates the risk for the occurrence of CVD conditions including CAD, stroke, atrial fibrillation, and heart failure. Thus, in individuals with DM, CVD continues to be the leading cause of mortality [46]. Consequently, understanding the complex pathophysiology of DM is essential to mitigating its effects. A brief comparison of the pathophysiological mechanisms underlying Type 1 and Type 2 DM is illustrated in Fig. 3.

### 4.2 Type 1 Diabetes Mellitus (T1DM)

T1DM is typically classified as a genetic immune system disorder that affects individuals across different age groups, including preadolescents, young adults, and older patients. The underlying mechanism behind T1DM development is the loss of pancreatic β cells, primarily driven by T-lymphocytes [47]. This immune response leads to inflammatory lesions known as insulitis, where T-cells and other immune cells infiltrate the pancreatic islets. Thus, it can be implied that islet infiltration is a β-cell-driven procedure because of the fact that insulitis is only present in β-cell islets [48]. The presence of T-cells is crucial as they lead to inflammation and contribute to the subsequent destruction of β cells within these islets [48]. The presence of specific genes can inhibit the immune system’s ability to distinguish between self and non-self, leading to autoimmune diseases including T1DM [47]. The genes, which are typically associated with T1DM, are often known as human leucocyte antigen (HLA), and belong to the major histocompatibility complex (MHC) region. It is recognized that 40%–50% of the genetic risk of T1DM derives from HLA complex polymorphic alleles. The *HLA *gene locus is categorized into Class I and Class II. The *HLA* alleles associated with T1DM belong to Class II, which are crucial for antigen presentation to T helper lymphocytes. Any substitution to the peptide-binding regions of Class II alleles can significantly affect the binding affinity for autoantigens, potentially leading to either a reduction or enhancement in the autoimmune response [49].

Numerous studies have documented abnormalities in the composition of leukocytes associated with T1DM. These include the natural killer T (NKT) cells, Dendritic cells, CD45R-subpopulations, CD4 and CD8 T-cells. However, the causes and their effects on disease progression are still under investigation [48]. Pathological responses to insulin deficiency include significant impacts on protein, glucose, and lipid metabolism. Specifically, insulin deficiency affects glucose metabolism, leading to metabolic disorders that may manifest as glucosuria, polydipsia, and polyphagia. In uncontrolled T1DM, an escalation of free fatty acids (FFA) in the plasma occurs due to the fast mobilization of triglycerides (TG). Insulin deficiency further leads to hypertriglyceridemia and increases the rate of proteolysis, raising plasma amino acid concentrations. Additionally, hyperglycemia in T1DM is exacerbated by hepatic and renal gluconeogenesis of glucogenic amino acids [45].

### 4.3 Type 2 Diabetes Mellitus (T2DM)

In T2DM, a progressive rise in plasma glucose levels results from the impairment in both insulin secretion and action [50]. Since it is a multi-factorial disease, it has been quite tough to comprehend its pathophysiology. However, T2DM can be classified as a genetic condition. Various genes are in control of the different chemical steps in the procedure of insulin secretion, insulin action and β-cell function. These genes are also essential for the synthesis of necessary enzymes. Any genetic impairment can trigger a cascade of metabolic reactions such as blocking insulin action (which further interferes with cellular glucose uptake), a rise in liver glucose production, prevention of uptake of fatty acids (FAs) and glucose, and the rise of TG breakdown. [47]. The deterioration of insulin secretion includes both glucose toxicity and lipo-toxicity, and can lead to a decline in β-cell mass when left untreated, as demonstrated in animal experiments. The progression further impairs the long-term control of glucose levels in the blood [51]. Various, factors are responsible for the loss of the β-cells and these include glucolipotoxicity and amyloid deposition that progresses in β-cell apoptosis by oxidative and endoplasmic-reticulum stress [52].

Other organs have a huge role in the pathophysiology of T2DM [50]. Hepatic glucose overproduction occurs due to the liver’s insulin resistance coupled with elevated circulating glucagon and increased liver sensitivity to glucagon. Furthermore, insulin can cross the blood brain barrier (BBB) where it can suppress hunger by regulating the expressions of neuropeptides regulating food intake. Another complication from excess circulating FFAs is that of resistance of adipocytes to insulin’s anti-lipolytic effect. This chronic surge in FFAs contributes to several metabolic disturbances including gluconeogenesis, IR in skeletal muscle and the liver, and impaired insulin secretion [53].

In patients with nonalcoholic fatty liver disease (NAFLD), which is commonly associated with T2DM, IR leads to an increased accumulation of FFAs in the liver. This accumulation not only exacerbates NAFLD through chronic liver cell stress but also initiates a cycle of further lipid buildup. Additionally, obesity, a significant factor in the development of T2DM, reduces the levels of adiponectin, a hormone that normally inhibits proinflammatory cytokines such as tumor necrosis factor alpha (TNF-α) and interleukin 6 (IL-6). The reduction in adiponectin levels results in increased production of these cytokines, leading to inflammation in adipocytes and further exacerbating IR [54]. This inflammation directly contributes to obesity-induced IR through the I-kappa-B kinase β (IKKβ)/nuclear factor kappa-light-chain-enhancer of activated B cells (NF-κB)-dependent pathway. Moreover, obesity increases the number of adipose tissue macrophages (ATMs). These macrophages become more active and express higher levels of proinflammatory genes, intensifying the inflammation associated with obesity. ATMs not only contribute in the inflammation but also play a role in lipid metabolism. Research on different immune cells in adipose tissue has shown that obesity activates pro-inflammatory immune cells while inhibiting anti-inflammatory ones, disrupting the balance between these cell types in visceral fat and leading to systemic IR in the liver and muscles [55]. Thus, DM management guidelines recommend at least a 5% weight loss in overweight and obese patients. For efficient DM management, it was paired with a customized diet, more exercise, and behavioral therapy [6].

Multiple genetic studies comparing various geographic and ethnic groups have indicated that the risk of acquiring T2DM is not uniform across all demographic groups. These studies reveal significant variations based on lifestyle, age, sex, ethnicity, family history, and body fat distribution [56, 57]. Similarly, a meta-analysis revealed that first-generation migrants in Europe experienced a higher risk of T2DM compared to native Europeans. The highest risk was observed among South Asians, followed by migrants from the Middle East and North Africa, sub-Saharan Africa, the Western Pacific, and South and Central America, underscoring the role of ethnicity in T2DM prevalence. Lifestyle interventions among these immigrants have been shown to result in a modest but statistically significant reduction in their glycated hemoglobin (HbA1c) levels and improvement in the knowledge and practices related to DM self-management [58]. Another study also produced similar results indicating that lifestyle weight-loss interventions in overweight and obese adults with T2DM can improve HbA1c levels, with the impact varying by ethnicity. The most significant benefits were observed among Whites, followed by Asians [58]. Beyond ethnic variations, the biological mechanisms that contribute to T2DM also differ by sex. Males are marginally more prone to being diagnosed with T2DM, often at a younger age and with a lower BMI, while females are more likely to experience impaired glucose tolerance, with a history of gestational DM serving as a key risk factor. Hormonal factors, such as polycystic ovary syndrome (PCOS), early menopause, and elevated testosterone levels, increase the risk for females, whereas low testosterone levels heighten the risk for males. Even though females with T2DM face a higher relative risk of cardiovascular complications and mortality compared to males, the absolute risk is still greater in males [59].

### 4.4 Pathophysiology of Obesity

The correlation between obesity and DM, typically T2DM, is well known and has been studied for many years. Obesity plays a crucial role in the occurrence of T2DM [60]. It is a chronic condition in which hypertrophy and hyperplasia of fat cells occurs. The several clinical conditions that are associated with obesity include heart disease, DM, liver disease, and various cancers. These diseases are the outcomes of either the increased weight of fat cells or the excess secretion of FFAs from enlarged fat cells [60]. It is this increase in plasma FFAs that dominates in obesity. Fat distribution also typically influences IR along with elevated secretion of FFA from visceral fat cells into the portal venous system [60, 61]. Two features are typically important for obesity to produce T2DM. The first is impaired insulin response in skeletal muscle. The second is the inability of β-cells to secrete the necessary levels of insulin required to maintain the normal blood glucose levels. Both factors contribute to the progression of IR to T2DM [62].

Obesity is a major factor in the occurrence of metabolic disorder. Adipose tissue regulates metabolism by the secretion of non-esterified fatty acids (NEFA), glycerol, and proinflammatory cytokines. It also regulates hormones like leptin, adiponectin. The secretion of NEFAs is recognized as the single most crucial factor in the regulation of insulin sensitivity. Elevated NEFA levels have been observed in both obesity and T2DM, and are associated with IR in both conditions. An escalation in plasma NEFA triggers IR within hours in humans [63]. Chronic hyperglycemia and an increased NEFA level can further contribute to β-cell dysfunction [60]. Thus, the association of hyperglycemia with IR and β-cell dysfunction is very clear [64]. The hyperplasia and hypertrophy of adipose tissues caused by obesity are often driven by the excessive release of pro-inflammatory cytokines from these tissues, known as adipokines, including TNF-α, IL-6, and interleukin-3 (IL-3). This eventually leads to IR, which can arise either by direct interference with the canonical insulin signaling pathway or by amplified activation of other inflammatory pathways [65].

### 4.5 Hypertension

One of the crucial risk factors for the development of DM is hypertension [66]. In a prospective cohort research, it was determined that hypertensive patients experience a greater risk of developing DM in comparison to patients with normal BP [66, 67]. A 2010 study involving 1601 revealed a substantial co-occurrence of DM, hypertension, and obesity. Additionally, 18.1% of patients had obesity, hypertension and DM, 16.1% had both hypertension and DM, 15.4% of patients had obesity and hypertension, 12.7% had obesity and DM [68]. There is a close association between reduced glucose tolerance and high BP. High BP is more common in both T1DM and T2DM than in non-diabetic patients. However, the causes for this increased prevalence vary by DM type [69]. For the effective management of hypertension and DM simultaneously, it is important to understand their pathophysiology. Since the two conditions usually co-exist, it can be understood that they share similar pathophysiological mechanism of action [70].

A 2011study determined that hypertension is a primary factor in cardiovascular events in diabetic patients [71]. Extensive data collected from various studies have shown that strictly controlled BP potentially decreases the risk of CVD in diabetic patients compared to the non-diabetic counterparts [72]. In the pathogenesis of hypertension and DM the renin angiotensin aldosterone system (RAAS) plays a crucial role. Angiotensin II hormone binds with angiotensin type 1 (AT1) receptor resulting in vasoconstriction, a rise in sodium reabsorption, and the stimulation of the release of aldosterone from adrenal cortex, which cumulate in the stimulation of sodium reabsorption, ultimately causing an escalation in BP [70]. Angiotensin II causes vascular injury by several ways such as vasoconstriction, inflammation, cell growth, and production of oxidative stress. It also regulates vascular inflammation by inducing the secretion of cytokines and pro-inflammatory transcription factors like nuclear factor κB (NF-κB) [73]. It has been shown in numerous studies that in CHD or in the case of its risk factors, the plasma markers for oxidative stress typically increase. The oxidation of weak cell membrane unsaturated lipids may stimulate diverse signal transduction pathways to drive various adverse events associated with atherosclerosis pathophysiology [74]. Hypertensive diabetic patients typically present uncommon characteristics when compared to non-diabetic hypertensive patients. These characteristics include a high propensity for proteinuria, orthostatic hypotension, high salt sensitivity and volume expansion, loss of nocturnal dipping of BP, and isolated systolic hypertension. The majority of these factors are associated with an increased risk of CVD [75].

### 4.6 Dyslipidemia

Dyslipidemia is a condition that promotes atherosclerosis. It is a complex condition and a critical risk factor known to influence CVD [76]. Dyslipidemia associated with obesity is characterized by the presence of elevated FFAs and TG, lower high-density lipoprotein cholesterol (HDL-C) levels along with dysfunction of high-density lipoprotein (HDL), a normal to slight rise in LDL-C levels and rise in small dense low-density lipoproteins (LDLs) [77]. Dyslipidemia has been characterized as a major cardiovascular risk factor in T2DM. A study illustrated LDL-cholesterol levels of more than 2.6 mmol/L in 84% of male and 88.7% of female diabetic patients. Thus, it is evident that diabetic dyslipidemia is a very common event [78]. A study has also shown an increased production of very low-density lipoprotein- apolipoprotein B (VLDL-apoB) by the liver as a dominant characteristic in this disease [79]. In patients with DM, an increase in postprandial VLDL and apoB100 production typically occurs due to insulin resistance. ApoB48 production has also been found to rise in T2DM patients, and is correlated with plasma insulin levels [80]. Several metabolic processes are responsible for this increase in pro-atherogenic apoB-rich lipoproteins and HDL dysfunction. Studies have also shown that increased plasma levels of apolipoprotein B (apoB) [81] and small dense LDL [82] are crucial determinants associated with atherosclerosis in T2DM [79].

### 4.7 Diabetic Cardiomyopathy (DCM)

Diabetic cardiomyopathy (DCM) is a common condition that typically occurs as a result of hyperglycemia influenced by IR syndrome, which in turn causes left ventricular hypertrophy [83]. This condition is also associated with diastolic dysfunction, and is more likely to occur in patients with hypertension or myocardial ischemia [84]. Hyperglycemia, hypertension, and impaired endothelial function are some of the factors that influence the disease mechanism and can potentially compromise myocardial blood flow. Another contributing factor might be atherosclerosis [85]. The pathophysiology of DCM is multifactorial [84]. In diabetic patients, increased glucose levels can impair endothelium physiological characteristics leading to endothelial dysfunction including decreased fibrinolysis, increased permeability, and leukocyte adhesion [86]. Similarly, increased FFA levels associated with hyperglycemic conditions and/or IR causes lipotoxicity, which can also be a potential factor in DCM due to FFA and their products inducing myocardial oxidation. These cumulative insults can lead to development of the disease [83]. Reactive oxygen species (ROS) production from both mitochondria and cytosol are also significant factors in DCM development. Specifically, ROS can induce lipid and protein impairment by oxidation. Chief among these proteins are DNA repair enzymes, which may exacerbate the situation by driving DNA damage [86].

### 4.8 Cardiovascular Autonomic Neuropathy (CAN)

CAN is a condition that causes impairment in the autonomic nerve fibers which innervate the heart. Specifically, abnormalities may lead to changes in both vascular dynamics and heart rate control [87, 88]. CAN results from complex, intertwined interactions between disease duration, age-related neuronal death, glycemic control levels, as well as both systolic and diastolic BP. Hyperglycemia has a crucial role in activating several biochemical pathways connected to the metabolic and redox cell states. This, in combination with impairment in nerve perfusion, influences the development of diabetic neuropathy [89]. Hyperglycemia is also known to increase the production of mitochondrial free ROS thus generating oxidative damage to microvasculature structure which supply peripheral nerves [88]. Elevated plasma glucose increase protein glycation, resulting in an excess of AGEs. Studies have shown that AGE accumulation in collagen tissue corelates with the severity and extent of peripheral and autonomic nerve abnormalities in diabetic individuals, often preceding clinical symptoms [90]. Furthermore, a multicenter study involving 400 individuals with diabetes found that 25% of the patients exhibited these symptoms, indicating the presence of CAN [91]. These findings suggest that CAN is relatively prevalent among individuals with diabetes.

### 4.9 Myocardial Infarction (MI) Associated to DM

In pathology, MI is defined as myocardial cell death caused by prolonged ischemia. Myocardial ischemia arises from an imbalance between the supply of oxygen and its demand, representing the first step in the development of MI [92]. Individuals with T2DM are at a heightened risk for all three major cardiovascular events compared to those without the condition. This risk is similar for both men and women regarding stroke (1.8 times higher) and heart failure (2.7 times higher). However, when it comes to MI, women exhibit a significantly greater risk than men, with incidence rate ratios (IRRs) of 2.58 (95% CI: 2.22–3.00) for women versus 1.78 (95% CI: 1.60–2.00) for men, indicating a significant interaction (*p* < 0.0001) [93]. Furthermore, acute hyperglycemia is associated with increases to mortality rates, infarct size, and of left ventricle functional damage following acute myocardial infarction (AMI). Hyperglycemia stimulates platelet aggregation and coagulation [94] and induces the rapid suppression of flow-mediated vasodilatation, most likely by the escalated production of oxygen-derived free radicals. Similarly, a rise in oxidative stress also interferes with vasodilation mediated by nitric oxide (NO) and decreases the coronary blood flow at microvascular level [95]. Beyond the previously mentioned mechanisms, several other factors are responsible for the pathogenesis. Notably, the prevalence of DM in AMI patients is well-documented [96]. In a population-based study, one out of every four hospitalized AMI patients were also diagnosed with DM [97].

## 5. Risk Factor for DM and CVD

**Fig. 4.**
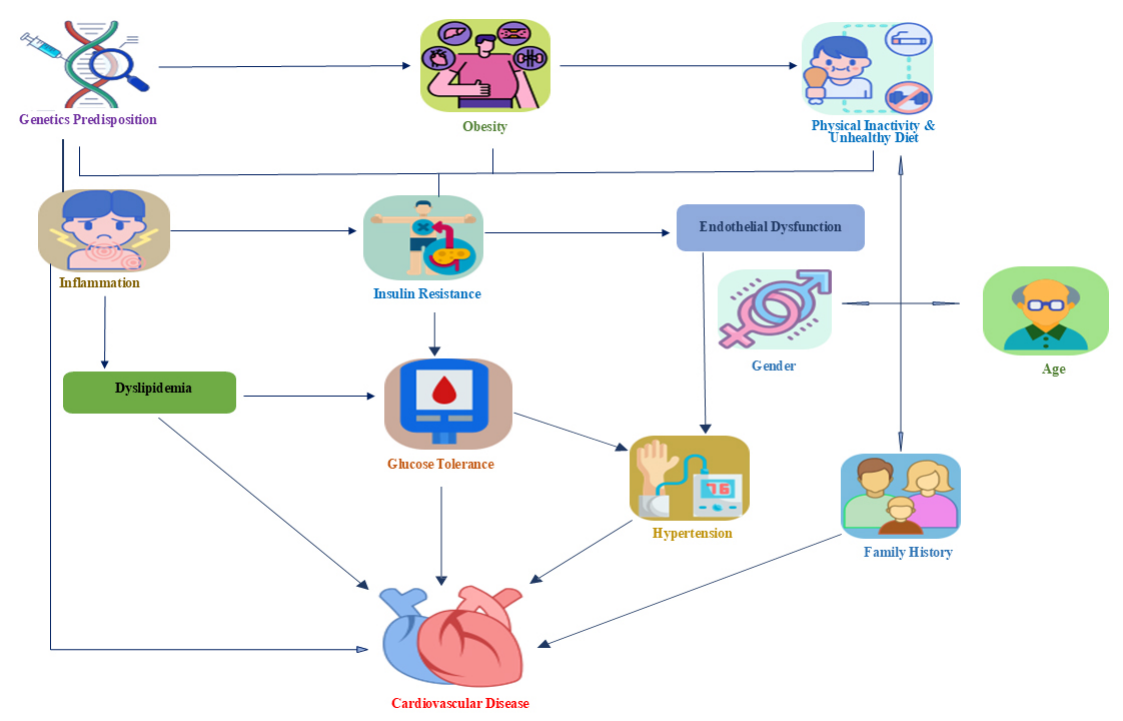
**Interactions of risk factor for diabetes mellitus (DM) and cardiovascular disease (CVD)**. This figure visually represents the complex interplay of modifiable and non-modifiable risk factors that contribute to the development of DM and CVD. This highlights their significance in screening and intervention strategies to mitigate potential CVD risks.

There is a widely accepted agreement that traditional lifestyle factors, such as unhealthy diet (excessive sodium and meat consumption, and inadequate intake of fruits, nuts, and vegetables), lack of physical activity, and smoking, are closely associated with the rising burden of CVD. Other lifestyle factors that significantly contribute to metabolic diseases include high BP, obesity, dyslipidemia, and dysglycemia (pre-diabetes and T2DM), which serve as independent precursors to CVD. Additional non-modifiable risk factors, such as age, male sex, genetic predisposition, and family history of CVD should be taken into account during screening and when considering interventions for potential CVD risk [98]. The complex interactions of both modifiable and non-modifiable risk factors contributing to the development of DM and CVD are shown in Fig. 4.

### 5.1 Physical Inactivity and Unhealthy Diet

Diabetic individuals are less likely to engage in regular physical activity compared to those without DM. This lack of physical activity is linked to higher mortality rates among individuals with DM. While regular exercise generally promotes good health, it is advisable for individuals with DM to undergo a medical assessment before initiating any exercise regimen. Various factors such as foot ischemia, loss of protective sensation, risk of vitreous hemorrhage, resting tachycardia, and postural hypotension are linked to specific diabetic vascular complications. These factors must be taken into consideration when developing exercise plans to ensure patient safety. Research on the impact of diet on DM development has produced mixed results regarding the risk associated with certain nutrients. Nonetheless, it’s evident that a diet rich in saturated fats is linked to unfavorable cardiovascular risk factors, regardless of DM status. The American Diabetes Association (ADA) doesn’t endorse a singular “diabetic diet”. However, advocates for an individualized strategy based on extensive patient evaluation and therapeutic goals [99]. A study conducted in 2016 demonstrates that an increase in physical activity significantly lowers risk of both CVD and T2DM. Specifically, an increase of 11.25 metabolic equivalent of task hours per week of activity for an otherwise inactive person, produced a 23% reduction in cardiovascular mortality and a 26% decrease in risk for DM in DM risk, regardless of body weight [100].

### 5.2 Age, Gender and Family History

Elderly individuals are more susceptible to CVD due to age-related changes in the structure and function of the heart and blood vessels. While males face a greater risk of heart disease compared to females before menopause, postmenopausal females have a similar risk level to males (Fig. 4). Cardiac death often occurs suddenly, and without prior cardiovascular symptoms. It’s worth noting that the risk of premature death from sudden cardiac death is approximately 1 in 9 for males and 1 in 30 for females. This underscores the importance of public health initiatives aimed at preventing sudden cardiac death. A family history of stroke or CHD is also a significant, unchangeable risk factor for CVD in future generations (Fig. 4). It is highly advisable for individuals with such nonmodifiable risk factors to undergo regular check-ups [101].

### 5.3 Tobacco Smoking

Smoking tobacco substantially raises the risk of developing T2DM and its associated complications, especially among heavy smokers. Furthermore, research on people with DM reveals that exposure to secondhand smoke increases the chances of developing CVD, microvascular issues, and premature mortality. The expected burden of diseases caused by smoking may affect females more than males. This is not only due to changes in the prevalence of smoking and smoking habits over the past century but also because females may have a stronger link between long-term smoking and the risk of CVD when compared to males. The initial evidence indicating that female smokers might face a higher relative risk of CVD came from a study conducted in Denmark, which revealed that female smokers had a 50% higher risk of CHD compared to male smokers. Subsequent research indicates that smoking is more strongly linked to CHD risk in females than males, especially among those who smoke over 20 cigarettes daily [102]. On the contrary, quitting smoking is linked to notable reductions in conditions like microalbuminuria, high BP, abnormal lipid levels, and IR [103].

### 5.4 Dyslipidemia and Obesity

Individuals diagnosed with DM have an increased susceptibility to dyslipidemia. This correlation is partially due to the enhanced release of FFAs in insulin-resistant adipocytes. Elevated FFA levels promote TG synthesis, consequently triggering the release of apoB and VLDL cholesterol [12]. The risk of CVD remains high in cases of dyslipidemia, which are characterized by an atherogenic profile, including elevated levels of VLDL, TG, and LDL, along with reduced levels of HDL. Furthermore, diabetic hyperlipidemia can increase atherosclerosis, and the combination of obesity with DM is associated with a significant increase in the rates of CVD-dependent illness and death (Fig. 4). In cases of obesity, visceral fat accumulation triggers inflammation, a major factor in DM complication [104]. Obesity significantly increases the risk of heart failure, a link that is not fully explained by the typical cardiovascular risks associated with obesity. Those with obesity face twice the risk of heart failure compared to those with normal weight. Data from the Framingham Heart Study indicates that each one-unit increase in BMI is associated with a 5% increase in heart failure risk for males and 7% for females, even after adjusting for other heart risk factors. This risk extends across all BMI levels. A meta-analysis on obesity and heart failure found that every 10 cm increase in waist circumference (WC) is linked to a higher incidence of heart failure, with this association being particularly pronounced in individuals within the overweight BMI range [105]. Moreover, obesity results in elevated levels of leptin and inflammatory indicators like C-reactive protein (CRP), exacerbating vascular and myocardial damage. Individuals with a BMI exceeding 40 are significantly more likely to be diagnosed with DM (odds ratio (OR) = 7.37, 95% CI: 6.39–8.5), hypertension (OR = 6.39, 95% CI: 5.67–7.16), and hypercholesterolemia (OR = 1.88, 95% CI: 1.67–2.13) when compared to those within a normal weight range [106]. A meta-analysis consisting of randomized controlled trials (RCTs) and prospective cohort studies evaluated the effects of MedDiet and the risk of occurrence or death from CVD, CHD, stroke, and MI in individuals with DM. Pooled analyses from RCTs shows that the MedDiet effectively lowers the risk of total CVD and MI incidence in these individuals [107].

### 5.5 Hypertension

Modifiable risk factors contribute to CVD, including many cases that result in fatalities. Notably, hypertension has significant global impact, exerting a greater influence on the incidence of strokes than MI [108] (Fig. 4). The increase in hypertension cases is strongly tied to dietary habits, especially high sodium intake. The large-scale Prospective Urban Rural Epidemiology (PURE) study confirms sodium consumption as a major contributor to both hypertension and cardiovascular disease [109]. In individuals with mild to moderate hypertension, the greatest risk is observed among those diagnosed with dyslipidemia, DM, and left ventricular hypertrophy. In elder hypertensive individuals, it’s common to detect signs of organ damage, including impaired renal function, silent MI, strokes, transient ischemic attacks, retinopathy, and PAD. The Framingham study revealed that at least 60% of older males and 50% of older females with hypertension had one or more of these complications [110]. A meta-analysis involving one million adults across 61 observational studies revealed a correlation between BP levels and the risk of death from ischemic heart disease and stroke. Specifically, it was observed that even at relatively low levels—115 mm Hg systolic blood pressure (SBP) and 75 mm Hg diastolic blood pressure (DBP)—there was a noticeable increase in mortality risk. A subsequent study found that for each increase of 20 mm Hg in SBP and 10 mm Hg in DBP, the risk of death from stroke and ischemic heart disease doubled. However, it was suggested that reducing SBP by 10 mm Hg and DBP by 5 mm Hg could lead to a significant decrease in the risk of stroke-related mortality by 40% and a 30% decrease in mortality from ischemic heart disease and other vascular-related causes [111]. Hypertensive disorders during pregnancy, encompassing conditions like gestational hypertension and pre-eclampsia, impact approximately 10% of pregnancies. Females with these conditions are, on average, twice as likely to develop CVD later in life compared to females with normal BP during pregnancy. This elevated risk may stem from a predisposition to CVD, the hypertensive disorder itself, or a blend of both factors [112]. Arterial hypertension affects over 60% of individuals with T2DM. This occurrence is directly correlated with: (1) heightened activity of the RAAS, (2) elevated levels of insulin leading to enhanced renal sodium reabsorption, and (3) enhanced sympathetic nervous system activity. Additionally, aging, obesity, and the development of kidney disease further increase the prevalence of hypertension. The combination of hypertension and DM significantly heightens the risk of CVD. While the presence of DM doubles the cardiovascular risk in males and more than triples it in females, hypertension increases this risk fourfold in diabetic patients [113].

### 5.6 Genetic Predisposition

The connection between genetic markers linked to atherosclerosis and the development of ASCVD in diabetic patients remains unclear. In an 8-year cohort study, various measures of subclinical atherosclerosis, such as coronary artery or abdominal aortic calcium score, common and internal carotid artery intima-media thickness, and ankle-brachial index, did not were not associated with a T2DM risk score based on 62 genetic loci. When comparing the fourth quartile of genetic predisposition risk score (GPS) with the first quartile, the risk of CVD rises with each additional genetic variant by 3%, with a maximal odds ratio of 1.47 [16]. The heritability of T2DM and CVD varies in the general population, but approximately 30% of the genetic component influencing risk can be attributed to individual differences. It remains unclear if common risk factors that predispose individuals to both ailments involve shared genetic elements or if T2DM disrupts pathways relevant to CVD’s development. Only a few strong genetic regions have been linked to T2DM and CVD, despite numerous Genome-Wide Association Studies (GWAS) research. While the prevailing causes of T2DM and the majority of CVD involve multiple genes, there are also instances where a single gene mutation can instigate these conditions. Specifically, certain gene mutations, when present in a heterozygous state, may contribute to familial forms of risk factors for cardiovascular ailments such as hypertension, high cholesterol levels, and T2DM. These genes, commonly associated with familial high cholesterol levels, may indicate a mechanistic involvement of cholesterol metabolism in the development of T2DM, given the condition’s association with impaired intracellular cholesterol transport [114]. T1DM becomes a chronic condition with the emergence of the first islet autoantibody, signifying ongoing immune-driven destruction of pancreatic-islet β cells. This destruction results from a complex interplay of genetic and environmental factors. Advances in human genetics have revealed over 50 genetic regions associated with susceptibility to T1DM. Apart from the significant influence of the HLA gene on chromosome 6p21, which contributes roughly half of the genetic risk, other identified loci exert only modest effects on the overall genetic predisposition to T1DM. The specific molecular mechanisms underlying these loci’s actions remain largely unknown [115]. Addressing risk factors is as important as generating methods and guidelines for the management of CVD and DM. The Treatment of Cardiovascular Risk in Primary care using Electronic Decision Support (TORPEDO) study consisting 60 Australian primary health care centers (40 general practices and 20 Aboriginal Community Controlled Health Services [ACCHSs]) assessed the impact of a Quality Improvement initiative. This program integrated point-of-care electronic decision support with audit and feedback tools, and found moderate improvement in screening and treatment levels in diabetic individuals [116].

## 6. Treatment Recommendations

### 6.1 Lifestyle Measures for Managing Diabesity

#### 6.1.1 Dietary Modifications

Improving diet quality, regardless of caloric restriction is applied, can reduce CVD risk and prevent T2DM in at-risk individuals. For patients with T2DM, any diet leading to significant weight loss may provide clinically meaningful glycemic control benefits [117]. While the American Diabetes Association (ADA) and the European Association for the Study of Diabetes (EASD) recognize low-carb diets as a therapeutic option, they consider the Mediterranean diet superior. Current meta-analyses show that the Mediterranean diet superior in terms of improving fasting glucose and lipid profiles and ranks among the top three diets for HbA1c levels, blood pressure, and weight loss. While low-carbohydrate diets are the most effective for reducing HbA1c levels and body weight, they also outperform low-fat diets in lowering fasting glucose, BP, and blood lipids [118]. Larger RCTs, like the Look-AHEAD trial, have shown that DM remission is linked to the extent of weight loss and inversely related to the duration of T2DM. Similarly, the UK-based DiRECT study compared a control group receiving standard DM care to a group in a structured weight management program. The weight management group achieved greater weight loss (10 kg vs. 1 kg) and a higher rate of DM remission (46% vs. 4%), with weight loss closely tied to improved DM outcomes [119]. Another meta-analysis suggests that low-carb diets (<26 energy% or <130 g carbohydrate [carbs]/day) may be more effective than low-fat diets for DM remission. After 6 months, more patients on low-carbohydrate diets reached HbA1c levels below 6.5%, though the difference becomes insignificant when factoring in medication usage or longer intervention periods [118]. Recent studies explored the potential of modulating gut microbiota through diet to influence satiety and insulin sensitivity pathways. The study suggests that the Mediterranean diet may effectively promote a balanced gut microbiome [119]

#### 6.1.2 Physical Activity

Regular physical activity enhances cardiometabolic and musculoskeletal health, aids in weight management, boosts cognitive and psychosocial function, and is linked to lower mortality from cancer and DM [120]. A recent systematic review of 53 studies, including 66 lifestyle intervention programs, found that diet and physical activity promotion programs significantly reduce T2DM incidence, body weight, and fasting blood glucose (FBG), while improving cardiometabolic risk factors compared to usual care. Less intensive interventions were also effective, and strict guideline adherence was linked to greater weight loss. Another meta-analysis of 10 trials in youth under 18 with T1DM showed significant HbA1C improvements in those who exercised, especially with more frequent and longer sessions that combined aerobic and resistance exercise. In adults, regular physical activity was linked to reduced mortality [121]. Exercise programs should be personalized to fit patient preferences and co-morbidities. While exercise improves biochemical markers, it alone is insufficient for DM remission. A combined diet and exercise is recommended for optimal results. This conclusion is supported by the Malmö study, where patients following both diet and exercise modifications showed long-term benefits, with half of the T2DM patients achieving remission after 5 years. Improvements in glycemic control were linked to both weight loss and increased fitness levels [119].

### 6.2 Current Antidiabetic Drugs

**Fig. 5.**
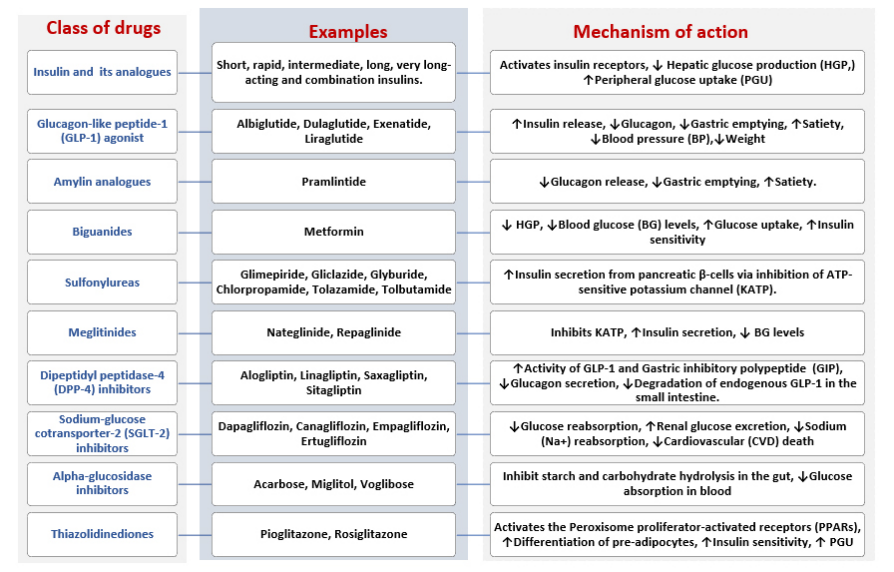
**Anti-diabetic drug classes and their mechanisms of action**. A summary of the various classes of oral antihyperglycemic agents (OHAs), also known as oral antihyperglycemic drugs (OADs), used in the treatment of type 2 diabetes mellitus (T2DM). This illustrates the pharmacotherapy options available for managing T2DM. ATP, adenosine triphosphate.

DM severely affects numerous metabolic activities and is driven by many biological mechanisms. Among the major two types of DM, the non-insulin dependent (NIDDM), T2DM has historically been treated with oral hypoglycemic agents (OHAs) also called oral anti-diabetic drugs (OADs). Hence, the use of various classes of OHAs, each with a distinct mode of action, is essential to optimize the pharmacotherapy. Fig. 5 illustrates an overview of the currently available and most widely used classes of anti-diabetic drugs. Each class targets distinct pathways to regulate blood glucose levels, contributing to improved glycemic control [49, 122, 123, 124, 125, 126, 127, 128].

**Fig. 6.**
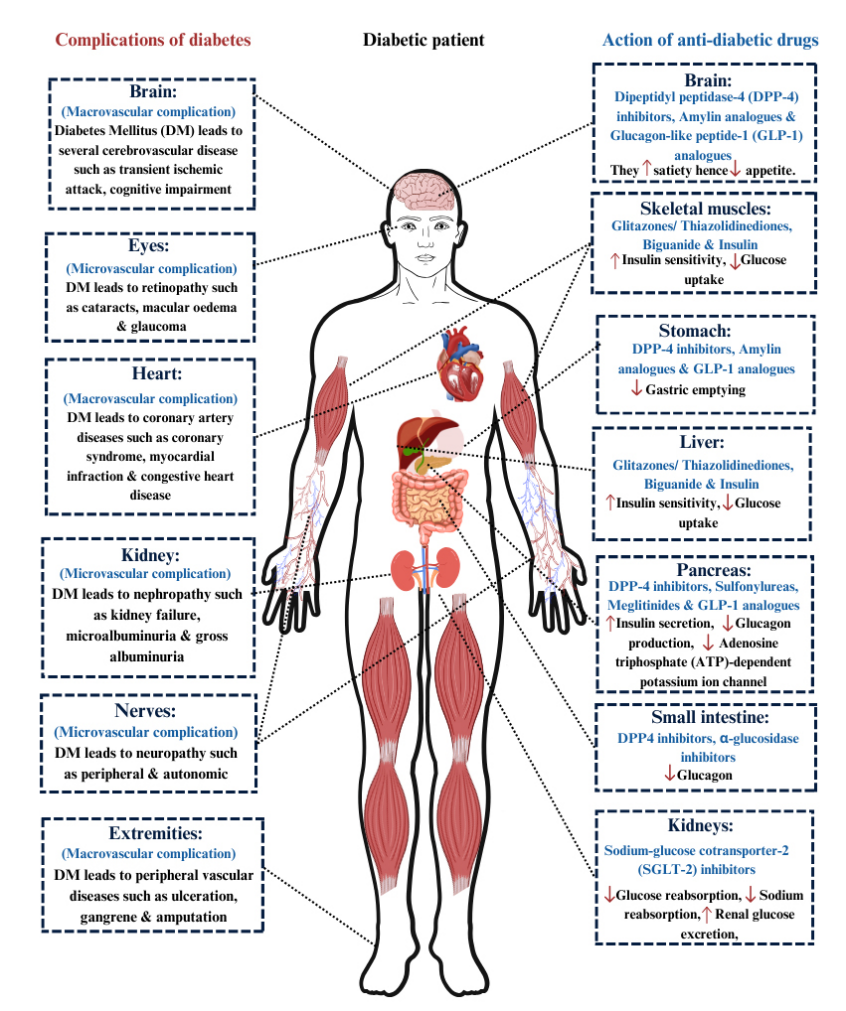
**Macro and microvascular complications of DM and the mode of action of anti-diabetic drugs on different organs of the body**. Fig. 6 portrays the complications of DM across different organs and tissues, highlighting the associated microvascular and macrovascular complications, as well as the therapeutic actions of anti-diabetic drugs. The left side details how DM impacts the brain, eyes, heart, kidneys, nerves, and extremities, leading to conditions such as retinopathy, CAD, nephropathy, neuropathy, and peripheral vascular diseases. On the right side, the diagram outlines how different classes of anti-diabetic drugs act on various organs of the body to alleviate these complications by modifying appetite, insulin sensitivity, glucose uptake, and other metabolic functions. CAD, coronary artery disease.

Many complications associated with DM across different organs and tissues. Particularly, there are associations between microvascular and macrovascular complications, as well as the therapeutic actions of anti-diabetic drugs. Some of these include the impact of DM on the brain, eyes, heart, kidneys, nerves, and extremities, leading to conditions such as retinopathy, CAD, nephropathy, neuropathy, and peripheral vascular diseases. Additionally, different classes of anti-diabetic drugs act on various organs of the body to alleviate these complications by modifying appetite, insulin sensitivity, glucose uptake, and other metabolic functions. These factors are illustrated in Fig. 6.

In addition to conventional anti-diabetic medications, recent advancements in pharmacotherapy for DM include the development of dual peroxisome proliferator-activated receptor (PPAR) agonists. These agonists facilitate increased utilization of lipids for energy production, rather than glucose, effectively reducing blood TG levels [129]. Among the PPAR agonists, the fourth class, known as Glitazars, interacts with both α and γ PPARs and is therefore referred to as dual PPARs [130]. Saroglitazar, the first clinically approved Glitazar, is an effective treatment for diabetic dyslipidemia and hypertriglyceridemia, especially in cases where statin therapy is unsuccessful [131]. Saroglitazar induces PPARα activation, which promotes hepatic fatty acid (FA) oxidation and decreases TG production and secretion. It’s hepatic action increases the transport of TG from peripheral tissues to the liver, resulting in a decline of the overall FA synthesis. Additionally, saroglitazar’s activity on PPARγ is associated with the regulation of insulin responsive genes, which improve β-cell function in pancreas and enhance insulin sensitivity. Thus, it exhibits both antidiabetic and antidyslipidemic effects [132].

### 6.3 Novel Therapeutic Agents

New therapeutic agents for DM that show promise for future use in effective DM management. Some of these agents are currently in phase 3 clinical trials, while others are still in the preclinical stage of development (Table 1, Ref. [133, 134, 135, 136, 137, 138, 139, 140, 141, 142, 143, 144, 145, 146, 147, 148, 149, 150]).

**Table 1.** **Novel anti-diabetic agents for DM management and adverse effects**.

Novel anti-diabetic agents	Biological activities	Adverse effects	References
Islet amyloid polypeptide (IAPP or amylin)	Regulates insulin action & ↓side effects of insulin monotherapy.	Mild to moderate gastrointestinal (GI) symptoms, including nausea, vomiting & headache.	[133]
	Human-IAPP analogs that can be used alongside insulin & other hormones (like glucagon) to ↑insulin monotherapy & support the creation of a fully automated closed-loop insulin delivery system for a true “artificial pancreas”.		
Pramlintide	Combined with preprandial insulin, it ↓postprandial hyperglycemia by ↓hyperglucagonemia & ↓gastric emptying. Pre-meal pramlintide with insulin also ↓ glycated haemoglobin (HbA1c) by 0.3–0.7% & ↓body weight by 0.4–1.4 kg.	Mild to moderate GI symptoms, including nausea, vomiting, & loss of appetite, were reported, alongside conditions like headaches, injuries, & sinusitis. Additionally, while none of the major studies indicated liver, kidney, or cardiac toxicity.	[134]
Antioxidant therapy-vitamins C, E, and β-carotene	It ↓blood sugar, ↓insulin resistance (IR) & ↓glycemic index in individuals with type ii diabetes mellitus (T2DM).		[135, 136]
Bromocriptine	It ↓blood glucose & ↓body mass index (BMI).	Headache, nausea, and vomiting.	[137, 138]
FBPase (fructose 1,6-bisphosphatase) inhibitors	The key enzyme in gluconeogenesis has become a valid molecular target for controlling glucose overproduction.	Liver carcinogenesis, liver hyperplasia, & liver hypertrophy.	[139]
Imeglimin	It ↑glucose-stimulated insulin secretion (GSIS) & preserves β-cell mass, while also ↑insulin action by ↓hepatic glucose output & ↑insulin signaling in the liver & skeletal muscle.	GI effects like nausea, vomiting, & abdominal pain.	[140, 141, 142]
	It ↑glycemic control by ↓HbA1c & ↓asting plasma glucose (FPG).		
Peroxisome proliferator-activated receptors (PPAR)	It ↓triglyceride (TG) level, ↓liver enzyme activity & ↓low-density lipoprotein (LDL) level.		[143]
GIP (glucose‑dependent insulinotropic polypeptide)	Significant role in T2DM & other metabolic disorders by ↑insulin response triggered by ↑post-prandial glycemia.	GI symptoms like Nausea.	[144, 145]
G-protein coupled receptor (GPCR 119)	It ↑glucose homeostasis through two mechanisms: directly ↑insulin release from β-cells & indirectly ↑glucagon-like peptide-1 (GLP-1) & ↑gastric inhibitory polypeptide (GIP) secretion from enteroendocrine cells.	GI symptoms like Nausea.	[145, 146]
FFA 1 (free fatty receptor‑1)	It impacts blood glucose levels through two mechanisms: indirectly ↑incretin hormones & directly ↑insulin release from pancreatic β-cells.		[145]
Melatonin	It ↑glucose regulation & ↑melatonin levels in the blood by ↑insulin secretion.	Headaches, nausea, dizziness convulsion, syncope, anxiety, depression, rashes, maculopapular rashes, constipation, & acute pancreatitis.	[145, 147]
Fucoidan	Maintain glucose homeostasis by ↓absorption in the gut & ↑muscle fiber utilization, preventing glycemia & lipedema. Its benefits in DM are attributed to its antioxidant properties & its role in ↓apoptosis, particularly in pancreatic beta cells, which preserves insulin secretion.	Abdominal distention, flatulence, meteorism & diarrhea.	[148, 149]
Quercetin	It shows antidiabetic properties by ↑oral glucose tolerance & ↑pancreatic β-cell insulin secretion. It ↓α-glucosidase & ↓dipeptidyl peptidase-4 (DPP-IV) enzymes, ↑GLP-1 half-life & ↑GIP half-life, while also ↓pro-inflammatory markers like ↓ interleukin- 1β (IL-1β), ↓ interleukin-4 (IL-4), ↓ interleukin-6 (IL-6), & ↓ tumor necrosis factor-α (TNF-α) level.	At doses exceeding 945 mg/m², quercetin may cause vomiting, hypertension, nephrotoxicity & lower serum potassium levels.	[150]

DM, diabetes mellitus.

### 6.4 Cardiovascular Implications of Widely Prescribed Oral Antidiabetic Drugs (OADs)

DM and CVD are interrelated, sharing several risk factors. Therefore, preventing or effectively managing DM can provide protection from CVD. Among the various classes of anti-diabetic agents, the biguanides (metformin) and the thiazolidinediones (rosiglitazone and pioglitazone) are noted for their potential cardiovascular protective effects. The United Kingdom Prospective Diabetes Study (UKPDS) stated that the treatment of overweight T2DM patients with Metformin was associated with a 39% decrease in MI, leading to its recommendation for treatment of overweight diabetic patients susceptible to CVD [151]. Despite having no effect on the HDL levels, Metformin considerably lessens the TG and LDL levels [152, 153]. Furthermore, Metformin is known for its insulin-sensitizing properties, enhances insulin sensitivity and helps renew endothelial function, which contributes to its anti-inflammatory and anti-thrombotic actions [154]. It exerts its thrombotic effects by inhibiting platelet aggregation and reducing fibrinogen levels, thereby offering cardiovascular benefits to diabetic patients [155, 156]. While metformin is effective in glycemic management, other OADs such as sulfonylureas and alpha-glucosidase inhibitors do not significantly improve the lipid profiles of diabetic patients [153]. On the other hand, thiazolidinediones (TZDs) act by lowering the plasma FFAs leading to the notable rise in HDL levels and a decrease in LDL levels [157, 158]. Beyond their impact on lipid profiles, TZD also reduce both SBP and DBP in diabetic patients with hypertension, underscoring their potential as cardioprotective agent [159, 160, 161, 162]. TZD includes the Pioglitazone and Rosiglitazone; while both agents increase the HDL levels, pioglitazone is uniquely effective in significantly reducing TGs [163]. As a result, combination therapy of Metformin and Pioglitazone provides enhanced cardiovascular protection [164].

###  6.5 Therapeutic Approaches towards Cardiovascular Diseases (CVD)

**Fig. 7.**
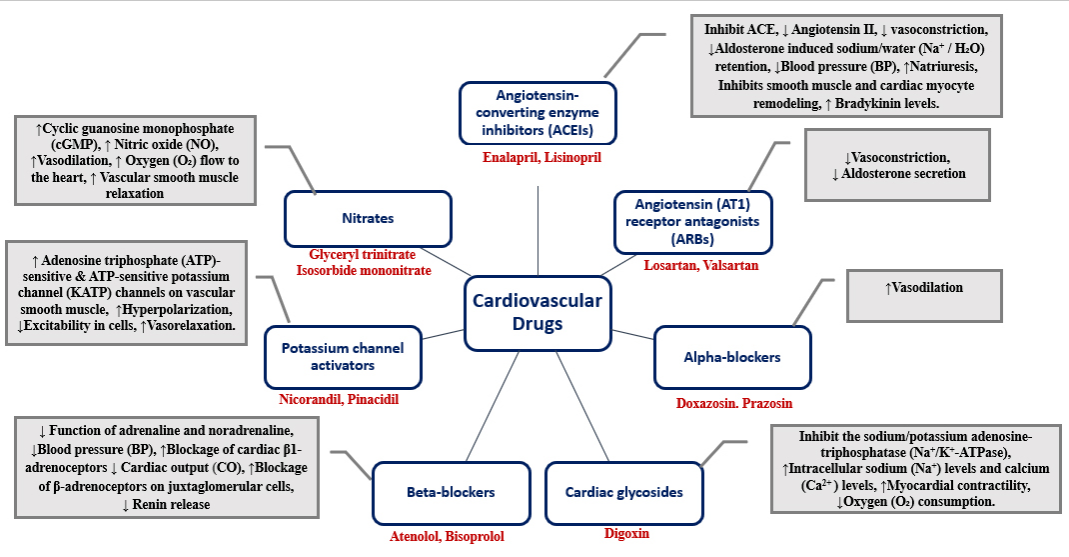
**Overview of cardiovascular drug classes and their mechanism of action**. Fig. 7 illustrates the diverse classes of cardiovascular drugs and their specific mechanisms of action within the cardiovascular system. It provides a comprehensive visual representation of the pharmacological strategies used to treat conditions ranging from hypertension to congestive heart failure, highlighting the targeted effects on either the heart or blood vessels [167, 168, 169, 170, 171, 172, 173]. ACE, angiotensin converting enzyme.

The increasing prevalence and high incidence of DM, coupled with rising obesity rates are making DM a greater cause of concern for CVD worldwide. Common among diabetic patients are decreased HDL and increased TG levels, alongside IR. Furthermore, DM is associated with impaired coagulation, inflammation, hyperglycemia, and glycation, all of which exacerbate the risk of CVD. Hence, a combination of CVD and DM reduces the overall life expectancy of an individual due to increased risk of recurrent disease, and CHF along with poorer surgical outcomes [165]. The risk of CVD is almost identical in diabetic and non-diabetic patients with a history of MI due to vascular damage as well as impaired glucose transport into the cells [166]. Cardiovascular drugs for the treatment of various conditions from high BP to CHF act on the heart or blood vessels and thus control the cardiovascular system. Fig. 7, depicts various categories of cardiovascular drugs each influencing heart function and vascular health through different biochemical pathways [167, 168, 169, 170, 171, 172, 173].

### 6.6 Diabetic Implications of Widely Prescribed Cardiovascular (CVD) Drugs

Among the array of widely used cardiovascular drugs, angiotensin converting enzyme inhibitors (ACEIs) and angiotensin (AT1) receptor antagonists (ARBs) are particularly beneficial in managing DM associated with macro- or microalbuminuria, both of which are closely linked to an increased risk of CVD [174]. The Heart Outcomes Prevention Evaluation (MICRO-HOPE) studies highlighted that ramipril, an angiotensin converting enzyme (ACE) inhibitor, significantly decreases the risk of diabetic nephropathy, stroke, cardiovascular fatality, revascularization, and ultimately overall mortality [175, 176]. Additionally, the Losartan Intervention for Endpoint reduction in hypertension (LIFE) study demonstrated that losartan, an ARB, significantly reduces cardiovascular fatality, stroke, and MI [177, 178].

### 6.7 Pharmacological Interventions of Cardiometabolic Risk in Obese Diabetic Patients

Obesity significantly increases the rates of mortality and morbidity associated with both DM and CVD [179]. Diabetic individuals typically exhibit a wider WC than their non-diabetic counterparts [180]. Similar to the potential cardioprotective effects of OHAs like metformin and TZD [151], newer diabetic therapies such as GLP-1 receptor agonists and SGLT-2 inhibitors also show promise in treating obese diabetic patients at risk for CVD [181, 182]. The Diabetes Prevention Study demonstrated the importance of weight loss in lowering DM risk and thus decreasing the probability of cardiovascular events [183]. Both GLP-1 and SGLT-2 produces weight loss, further demonstrating their efficacy in reducing deaths from CVD [181, 182]. A meta-analysis found that SGLT2 significantly decreased the likelihood of cardiovascular death or hospitalization from heart failure by 23% [96, 184] whereas GLP-1 appreciably lowered adverse CVD events and cardiovascular mortality by 10% and 13% respectively in T2DM patients [185].

### 6.8 Therapeutic Approaches towards Hypertension

Hypertension is one of the numerous factors contributing to the high prevalence of CVDs, which are the leading causes of mortality in diabetic patients. Antihypertensive ACE inhibitors decrease the likelihood that hypertensive patients to acquire DM by 11–34% [186, 187], highlighting their significant role in antihypertensive regimens to reduce CVD in in patients with DM. The hypertension optimal treatment (HOT) trial recommends maintaining a DBP below 85 mm Hg, which may reduce the prevalence of CVD [188]. For managing hypertension in diabetic patients, β-blockers have proven to be beneficial [189, 190], with atenolol specifically showing a significant reduction in morbidity and mortality from CVD in hypertensive diabetic patients, as demonstrated by the UKPDS study [191]. Additionally, fenoldopam, a calcium channel blocker, was used as the first-line treatment in the HOT trial, which demonstrated a decrease in major CVD events through DBP management in individuals with DM [192].

### 6.9 Therapeutic Approaches towards Hyperlipidemia (Dyslipidemia)

**Fig. 8.**
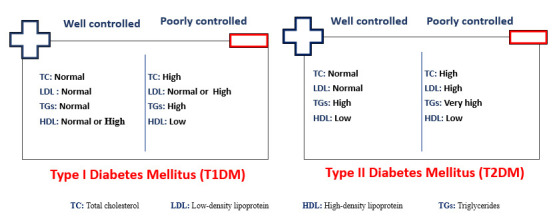
**Comparison of lipid profiles in well-controlled vs. poorly controlled DM**. This figure depicts the lipid profiles in well-controlled and poorly controlled cases of type 1 and type 2 diabetes mellitus, illustrating how lipid metabolism differs based on the level of diabetes management. The diagram emphasizes the impact of dyslipidemia on cardiovascular risk associated with diabetes. DM, diabetes mellitus.

Addressing dyslipidemia is expected to significantly reduce the incidence of complications such as CVD associated with DM, as it is a predominant risk factor [193]. In diabetic patients, dyslipidemia is characterized by hypertriglyceridemia, decreased level of HDL and increased levels of LDL particles (Fig. 8). These lipid abnormalities vary depending on the degree of diabetes management. As a result, the exacerbated lipoprotein level abnormalities due to dyslipidemia significantly elevate cardiovascular risk and mortality in diabetic individuals [194, 195, 196]. Fig. 8, compares the lipid profiles, between well-controlled and poorly controlled cases of T1DM and T2DM, highlighting the differences in lipid metabolism associated with each condition.

**Fig. 9.**
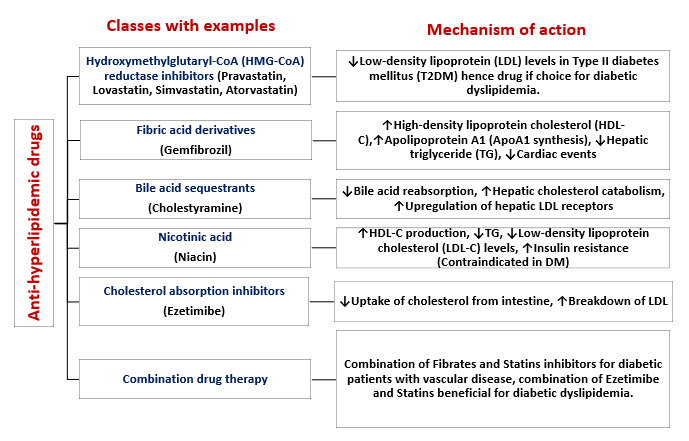
**Overview of anti-hyperlipidemic medications and their mechanism of action**. Fig. 9 provides a comprehensive overview of the various classes of anti-hyperlipidemic drugs, detailing their unique mechanisms of action and the specific ways they reduce lipid levels. It serves to illustrate the diversity and specificity of pharmacological approaches to lipid control in the context of diabetes management.

Various medication classes are employed to treat hyperlipidemia. These classes vary not just in terms of their mode of action but also in terms of the manner and degree of lipid reduction [196, 197]. The most popular class of anti-hyperlipidemic drugs, with examples and their respective mechanisms of action in managing dyslipidemia, particularly in diabetic patients have been presented in Fig. 9.

### 6.10 Diabetic and Cardiovascular Implications of Widely Prescribed Antihyperlipidemic Drugs

Statins, known as hydroxymethylglutaryl-CoA (HMG-CoA) reductase inhibitors, recognized for their ability to successfully lower LDL-C levels, are the first line drugs for treating diabetic dyslipidemia and preventing CVD [196, 197]. They minimize the risk of cardiovascular events and slow the onset of diabetic nephropathy. However, high-intensity statin monotherapy, particularly with rosuvastatin, have been found to be associated with a slight but significant risk of elevating HbA1c and fasting serum glucose levels in T2DM patients [198, 199, 200]. Despite this, the cardiovascular and mortality advantages of statin medication, even in pre-diabetic individuals, outweigh the minor increment in DM threats [197, 201] especially when used at moderate intensity [191].

Another class of anti-hyperlipidemic drugs, Fibrates are particularly effective in controlling TG levels and offer significant cardiovascular benefits in non-diabetic patients. However, fibrate monotherapy has not shown a positive impact in diabetic patients. In contrast, combining fibrates with statins significantly reduces the incidents of MI and revascularization in DM patients with dyslipidemia, especially in those with TG levels greater than 240 mg/dL and HDL-C levels below 34 mg/dL [202, 203]. Niacin, another commonly used anti-hyperlipidemic treatment, has no significant impact on cardiovascular complications, but is contraindicated in DM patient due to its hyperglycemic effects [89, 197, 198].

### 6.11 Therapeutic Approaches towards Cardiovascular Autonomic Neuropathy (CAN) in Diabetic Patients

Complications such as CAN in diabetic patients increase cardiovascular morbidity and mortality, including risks of cardiac arrhythmias, myocardial ischemia, DCM, stroke, and both intraoperative and perioperative cardiovascular (CV) instability [204]. Several studies such as the Diabetes Control and Complications Trial (DCCT) [205] have shown that better glycemic control reduces the incidence of CAN, particularly in patients with T1DM [206]. The French Multicenter study found that the prevalence of CAN, a prognostic indicator of microangiopathic complications, is correlated with obesity and the duration of DM, suggesting that adequate weight and glycemic management can lessen the risk of neuropathic complications [91]. Cardiac agents like β-blockers, aldose reductase inhibitors, ACE inhibitors, and satins, along with anti-hyperlipidemic drugs, are also capable of slowing the progression of CAN [87].

### 6.12 Clinical Trail

Various medicinal plants have been traditionally used to address numerous diseases, and many exhibits potential benefits for DM and cardiovascular conditions. Notably, plants like *Moringa oleifera Lam*., *Nigella sativa* L., *Panax ginseng* C.A.Mey., and *Panax quinquefolius* L. have shown effectiveness in combating DCM by significantly lowering FBG, TG, total cholesterol, and BMI. Table 2 (Ref. [207, 208, 209, 210, 211, 212, 213, 214, 215, 216, 217, 218, 219, 220, 221, 222, 223, 224, 225, 226, 227, 228]) provides an overview of clinical trials exploring the therapeutic potential of these and other medicinal plants in managing DCM and related metabolic disorders such as hypertension, and dyslipidemia. These studies focused on specific plant-based interventions and their outcomes, including improvements in lipid profiles, blood glucose reduction, BP regulation, and antioxidant activity. The results indicate promising pharmacological effects, suggesting that these plants could play a crucial role in the prevention and treatment of DCM and its associated conditions.

**Table 2.** **Clinical trials of medicinal plants for the treatment of DCM**.

Plant’s scientific names	Study model	Administered material	Duration	Subjects	Health condition of subjects	Dose	Pharmacological effects	Reference
*Allium sativum *L.	Randomized, single blind & placebo controlled	Tablet	24 weeks		Hypertensive patients with dyslipidemia	300 mg in doses of daily	It significantly improves serum lipid levels by ↓cholesterol levels, ↓TG levels, ↓LDL levels & ↑ HDL levels.	[207]
*Beta vulgaris *L*.*	Randomized, double-blind, placebo-controlled parallel trial	Naturally nitrate-rich beetroot juice	6 weeks	69	Hypercholesterolemic patients	250 mL once daily	It ↑flow-mediated dilation, ↓vascular stiffness & ↓platelet-monocyte aggregates.	[208]
*Camellia sinensis *(L.) Kuntze	Randomized, double blind, placebo-controlled trial	Tablet	2 months	100	Patients with T2DM	300 mg per day	It ↓TG levels, ↓total cholesterol levels, ↓BP & ↓oxidative stress.	[209]
*Commiphora mukul *Engl.*+ Commiphora myrrha *(T. Nees) Engl. *+ Terminalia chebula *Retz.	Randomized, double blind, placebo-controlled trial	Herbal combination capsule	3 months	86	Females with hyperlipidemic T2DM	200 + 200 + 200 = 600 mg, 3 times a day	It ↓FBG, ↓total cholesterol levels, ↓LDL levels & ↑HDL levels.	[210]
*Crocus sativus *L.	Randomized, double blind, placebo-controlled trial	Probiotic supplement with saffron	6 months	61	Patients with T1DM	Once daily	It ↓TG levels & ↓blood glucose levels.	[211]
	Randomized, double blind, placebo-controlled trial	Saffron consumption	8 weeks	70	Patients with T2DM	100 mg/day	It ↓TG levels, ↓LDL levels, ↓oxidative stress & ↑HDL levels.	[212]
	Randomized, double blind, placebo-controlled trial	Capsule	3 months	64	Patients with T2DM	15 mg (two pills per day)	It ↓TG levels, ↓LDL levels & ↑HDL levels.	[213]
*Ficus racemosa* L.	No placebo-controlled trial	Bark extract	15 days	50	Diabetic patients	100 mg two times	It ↓blood glucose levels.	[214]
	Randomized, double blind, placebo-controlled trial	Capsule	1 months	30	Patients with T2DM	1.2 g per day	It ↓blood glucose levels.	[215]
*Ginkgo biloba* L.	Randomized, double blind, placebo-controlled trial	Ginkgo biloba extract dietary supplement	90 days	60	Patients with T2DM managed with metformin	120 mg per day	It ↓fasting serum glucose levels, ↓blood glucose levels & ↓IR.	[216]
*Gymnema sylvestre* (Retz.) R.Br. ex Sm.	Randomized, double blind, placebo-controlled trial	Capsule	3 months	24	Patients with metabolic syndrome	300 mg twice daily	It ↓body weight, ↓BMI & ↓VLDL levels.	[217]
*Hibiscus sabdariffa* L. + *Olea europaea* L.	Randomized, double-blind, captopril-controlled trial	Capsule	8 weeks	134	Patients with grade 1 essential hypertension	Low-dose arm 2 two capsules of NW Roselle (1200 mg H. sabdariffa & 800 mg O. europea), high-dose arm 3 three capsules of NW Roselle (1800 mg H. sabdariffa & 1200 mg O. europea) twice daily	It ↓blood glucose levels & ↓TG levels.	[218]
*Momordica charantia* L.	Randomized, double blind, placebo-controlled trial	Capsule	12 weeks	96	Patients with T2DM	2380 mg twice a day	It ↓blood glucose levels.	[219]
*Moringa oleifera* Lam.	Randomized, double blind, placebo-controlled trial	Capsule	12 weeks	65	Patients with prediabetes	2400 mg/day	It ↓FBG levels & ↓HbA1c levels.	[220]
*Nigella sativa *L.	Randomized, double blind, placebo-controlled trial	Soft gel capsule	8 weeks	50	Patients with T2DM	500 mg twice a day	It ↓TG levels, ↓total cholesterol levels, ↓FBG levels & ↓BMI.	[221]
*Panax ginseng *C.A.Mey. + *Panax quinquefolius* L.	Randomized, double blind, placebo-controlled trial	Capsule	12 weeks	80	Patients with T2DM and hypertension	2.25 g 3 times daily	It ↓central SBP.	[222]
*Phoenix dactylifera * L.	Randomized, double blind, placebo-controlled trial	Date seed powder in the form of capsules	2 months		Patients with DM	8 g/kg in morning & evening after meal	It ↓blood glucose levels.	[223]
*Punica granatum *L.	Randomized, double blind, placebo-controlled trial	Tea bags	8 weeks	60	Patients with T2DM	5 g twice daily	It ↓FBG levels & ↓HbA1c levels.	[224]
*Trigonella foenum* graecum	A parallel randomized clinical trial	Powder	8 weeks	50	Patients with T2DM	5 g 3 times a day	It ↓FBG levels.	[225]
*Tecoma stans *(L.) Juss. ex Kunth + *Guazuma ulmifolia* Lam.	Randomized, double blind, placebo-controlled trial	Capsule	90 days	40	Patients with T2DM	400 mg	It ↓FBG levels & ↓HbA1c levels.	[226]
*Terminalia chebula *Retz.	Randomized, double blind, placebo-controlled trial	Capsule	12 weeks	60	Patients with T2DM	250 mg, 500 mg placebo twice daily	It ↓CVD risk & ↓hyperglycemia risk by regulating blood lipid & HbA1c levels.	[227]
*Zingiber purpureum *Roscoe + *Trigonellafoenum‐graecum* L.	Randomized, double blind, placebo-controlled trial	Capsule	8 weeks	33	Patients with T2DM	1 g 3 times a day	It ↓fasting blood sugar (FBS) & ↓HbA1c levels.	[228]

DCM, iabetic cardiomyopathy; TG, triglyceride; LDL, low-density lipoprotein; HDL, high-density lipoprotein; T2DM, type 2 diabetes mellitus; FBG, fasting blood glucose; BP, blood pressure; T1DM, type 1 diabetes mellitus; IR, insulin resistance; BMI, body mass index; VLDL, very low density lipoprotein; NW, NW Rosella; DM, diabetes mellitus; HbA1c, glycated haemoglobin; CVD, cardiovascular disease; SBP, systolic blood pressure.

## 7. Future Perspectives

CVD is the leading cause of morbidity and mortality among the in the growing worldwide diabetic population. Therefore, an intricate understanding of gene networks, intracellular pathways, and cell-to-cell communication mechanisms is essential. Such insights are crucial for advancing the development of new biomarkers and therapeutic strategies that are specifically tailored for treating CVD in individuals with T2DM [229]. The heart is deeply dependent on insulin signaling to manage its energy supply and metabolism, particularly in the processing of glucose and FAs. When IR occurs, it disrupts these vital functions leading to cardiac metabolic disturbances, autonomic dysfunction, subcellular signaling abnormalities, and activation of the RAAS. These disruptions collectively contribute to the development of diabetic cardiomyopathy, hypertrophy, fibrosis, and heart failure. As a result, IR significantly increases the risk of CVD, underscoring the importance of understanding the insulin–heart connection in developing effective treatments. As T2DM becomes more common, future research should emphasize both prevention and treatment, particularly by investigating new therapeutic targets. Studying specific signaling pathways and molecular mediators involved in insulin-stimulated glucose uptake and cardiac insulin signaling may lead to more effective treatments [230]. Current anti-hyperglycemic medications target various pathophysiological processes, including insulin secretion (sulfonylureas), peripheral glucose uptake (biguanides), and glucose reabsorption (SGLT2 inhibitors) [116]. Recent advancements include the approval of metformin and other contemporary antihyperglycemic regimens that combine three different medications. These triple combinations support the concept of a comprehensive multi-pathway therapy for T2DM. The diversity of T2DM is driving efforts to tailor therapies to optimize individual therapeutic responses and to minimize side effects [123]. Additionally, research is underway to develop oral insulin that mimics endogenous insulin by undergoing first-pass metabolism, thereby reducing hepatic glycogenolysis, gluconeogenesis, and potentially delivering more effective treatments [231]. However, data from previous studies suggest that metformin has minimal beneficial impacts on the cardiovascular outcomes of non-diabetic patients [232]. The ongoing VA-IMPACT study, which focuses on pre-diabetic patients with pre-existing CAD (Investigation of Metformin in Pre-Diabetes on Atherosclerotic Cardiovascular Outcomes, NCT02915198), is expected to provide further insights into metformin’s cardiovascular benefits by 2024 [232].

Preclinical studies have shown that the activation of cannabinoid receptors type 1 (CB1) in hepatocytes and skeletal muscle is associated with systemic glucose intolerance and insulin resistance. This has prompted the development of CB1 receptor antagonists and inverse agonists that specifically target peripheral tissues, avoiding central nervous system side effects. These compounds promote lipid mobilization, reduce triglyceride storage and glucose production, leading to weight loss, improved lipid profiles, reduced food intake, and enhanced insulin sensitivity. Such peripheral CB1 inhibition represents a promising approach for the safe and effective management of obesity and T2DM or diabesity [233]. Moreover, imeglimin, a novel mitochondrial bioenergetics enhancer, is the first OADs of its kind and shows promise in addressing primary DM complications, IR, and decreased insulin secretion by acting on the liver, muscles, and pancreas. Although imeglimin holds potential as a key treatment for DM, it is still undergoing Phase III trials in the US and Europe [234].

## 8. Limitations

There are many factors to consider when evaluating the limitations of various studies. Evidence suggests that females may receive less effective treatment compared to males following a cardiovascular event, leading to variations in risk factors during secondary prevention. For instance, US guidelines recommend administering high-intensity statins to survivors of MI. Since 2007, American females have had a decreased likelihood of receiving high-intensity statin prescriptions, even when they are prescribed statins. In 2014/2015, within the studied population, 9% (with a 95% confidence interval of 8 to 10%) fewer proportion of females compared to males received a prescription for high-intensity statins within 30 days after the event [235].

Two meta-analyses show statins increase DM risk, with 255 patients over 4 years experiencing one additional case of DM, a finding incorporated into the Food and Drug Administration (FDA) statin label [236]. Saxagliptin connection to an increased likelihood of hospitalization due to heart failure remains unexplained, thus caution is advised against its use in individuals with a history of CHF, particularly those with a history of MI. Alogliptin exhibits a slight rise in CHF-related hospitalizations, although not statistically significant, in the unstable angina and all-cause mortality among those under 65 years old taking Saxagliptin. However, the implications of these findings are uncertain, necessitating further investigation. Lixisenatide, a GLP-1 receptor agonist, demonstrated non-inferiority to placebo during a median follow-up of 2.1 years [237]. Sitagliptin and linagliptin are the preferred medications for individuals with T2DM and a higher risk of CVD, pending safety data [238]. Additional evaluations are warranted to explore Assessment of Cardiovascular Outcomes with Alogliptin versus Standard of Care trial. Similarly, there is limited evidence on the use of Linagliptin in individuals with a history of CHF, suggesting a need for careful consideration when prescribing these agents.

Furthermore, an additional analysis of the data hinted at a potentially higher risk of differences in treatment outcomes among different racial and ethnic populations. To illustrate, empagliflozin demonstrated a 32% reduction in ASCVD risk in the Asian cohort and a 48% increase in ASCVD risk in the black cohort compared to a placebo. Conversely, canagliflozin showed an 8% increase in ASCVD risk in Asian groups and a 55% reduction in ASCVD risk in black groups [239].

A recent study comparing various antidiabetic medications found that dipeptidyl peptidase-4 (DPP-4) inhibitors pose a greater risk of heart failure compared to other drug classes. Since there is no evidence supporting cardiovascular benefits from DPP4 inhibitors and some of them might even raise the risk of heart failure hospitalizations, they may not be the best option for diabetic patients who already have heart failure or are at risk of developing it, such as older patients, obese patients, or those with a long history of DM [240].

The present approach to treatment for individuals who have experienced AMI relies on findings from extensive RCTs and subsequent meta-analyses. These studies have proven that medications such as aspirin, β-blockers, ACE inhibitors, and statins improve outcomes following acute MI. While RCTs offer the most reliable evidence for evaluating the effectiveness and safety of medications, there are constraints in applying their findings due to the prolonged use of these drugs over an extended period [241].

Elderly patients should steer clear of long-acting sulfonylureas like glyburide and glimepiride. Caution should be exercised when using short-acting sulfonylureas such as gliclazide in patients prone to hypoglycemia, while patients with heart failure should avoid TZDs [102]. The primary constraints associated with insulin utilization among older adults encompass the likelihood of hypoglycemia, cost of insulin, the cost of blood glucose (BG) monitoring equipment, the requisite visual sharpness and manual adeptness for administering insulin injections, as well as the patient’s capability to balance dietary intake with insulin administration. Insulin analogs such as insulin glargine and insulin detemir are deemed safer than insulin-neutral protamine hagedorn (NPH) due to the inconsistent onset, substandard duration of efficacy, and increased risk of hypoglycemia associated with NPH [242].

Furthermore, as per prior research, measuring serum insulin levels directly is both expensive and not widely accessible in many developing regions. However, an alternative examination utilizing fasting TG and FBG is more cost-effective and readily available universally. Moreover, because exogenous insulin necessitates quantification, it can potentially disrupt the accuracy of the homeostatic model assessment for IR (HOMA-IR) index. Consequently, the current assessment of IR using the HOMA-IR index may not be suitable for diabetic patient undergoing insulin treatment. Conversely, the triglyceride–glucose (TyG) index, which relies on fasting TG and fasting glucose (FG), does require insulin quantification, thus making it applicable to a broader range of diabetic patients receiving insulin therapy [243].

Effective management of blood sugar levels holds greater significance among individuals diagnosed with T1DM, where disruptions in glucose metabolism significantly contribute to the heightened risk of ASCVD. Conversely, for those with T2DM, the risk of ASCVD stems from a multitude of factors such as dyslipidemia, hypertension, inflammation, IR, and coagulation disorders, among others, with glucose playing a relatively minor role in the enhanced risk.

Variations in other cardiovascular risk factors may explain why rigorous glycemic control led to a substantial decrease in ASCVD incidences in the DCCT, which focused on T1DM, while yielding minimal effects in trials targeting individuals with T2DM [244]. Early management of hyperglycemia, aiming to maintain HbA1c levels below 7%, benefits individuals with brief DM and low cardiovascular risk. However, this approach may not be as effective for older patients with prolonged hyperglycemia and heightened cardiovascular risk, suggesting the need for more stringent glycemic targets [1].

## 9. Conclusions

DM and CVD are inter-related because of the presence of shared risk factors. Therefore, prevention or at least proper treatment and management of DM can provide protection against cardiovascular events. The high prevalence of DM, coupled with risk factors like obesity, hypertension, dyslipidemia, genetic predisposition, tobacco smoking, sex, and family history increases the susceptibility of diabetic individuals to CVD. Additionally, conditions like CAN in diabetic patients further elevate the cardiovascular morbidity and mortality. A list of existing anti-diabetic medications has also been mentioned in this review, along with their respective mechanisms, such as Biguanide, GLP-1 agonists, DPP4-inhibitors, and SGLT-2 inhibitors. A number of medications are used to treat different risk factors of CVD such as TDZ which provides cardiovascular protection by decreasing the LDL levels in the blood plasma. Other drugs such as calcium channel blockers, statins and ACE inhibitors are also widely used as common treatment approaches. Similarly, the development of oral insulin that mimics the function of endogenous insulin appears to be a promising option. A detailed understanding of gene networks, intracellular pathways, and cell-to-cell communication mechanisms would allow for more prospective researches and development of treatment options. However, some limitations exist such as the use of DPP4-inhibitors must not be encouraged among diabetic individuals as they may increase the chances of cardiovascular events. To treat such a complex multifactorial disease, a multi-pathway treatment approach is required that not only mitigates the progression of CVD in diabetic patients but also prevents the development of the disease from its onset.

## Abbreviations

ACCHSs, Aboriginal Community Controlled Health Services; ACE, angiotensin converting enzyme; ADA, American Diabetes Association; AGE, advanced glycation end products; AMI, acute myocardial infarction; apoB, apolipoprotein B; ARBs, angiotensin receptor blockers; ASCVD, atherosclerotic cardiovascular disease; AT-1, angiotensin type 1; ATP, adenosine triphosphate; BBB, blood brain barrier; BG, blood glucose; BMI, body mass index; BP, blood pressure; Ca2+, calcium; CAD, coronary artery disease; CAN, cardiovascular autonomic neuropathy; CB1, cannabinoid-1; cGMP, cyclic guanosine monophosphate; CHD, coronary heart disease; CHF, congestive heart failure; CI, confidence interval; CO, cardiac output; CO₂, carbon dioxide; CRP, C reactive protein; CUPS, Chennai Urban Population Study; CVD, cardiovascular diseases; CVH, cardiovascular health; DBP, diastolic blood pressure; DCCT, Diabetes Control and Complications Trial; DCM, diabetic cardiomyopathy; DM, diabetes mellitus; DPP-4, dipeptidyl peptidase-4; FA, fatty acid; FBG, fasting blood glucose; FDA, Food and Drug Administration; FFA, free fatty acid; FMD, flow-mediated dilation; FPG, fasting plasma glucose; GIP, gastric inhibitory polypeptide; GLP-1, glucagon like peptide-1; GPS, genetic predisposition risk score; GSIS, glucose-stimulated insulin secretion; GWAS, Genome-Wide Association Studies; HbA1c, glycated haemoglobin; HDL, high-density lipoprotein; HDL-C, high-density lipoprotein cholesterol; HGP, hepatic glucose production; HLA, human leucocyte antigen; HMG-CoA, hydroxymethylglutaryl-CoA; HOMA-IR, homeostatic model assessment for insulin resistance; HOPE, Heart Outcomes Prevention Evaluation; HOT, Hypertension Optimal Treatment; HR, hazard ratio; HRV, heart rate variability; IAPP, islet amyloid polypeptide; IDDM, insulin dependent diabetes mellitus; IGT, impaired glucose tolerance; IL, interleukin; IR, insulin resistance; IRRs, incidence rate ratios; KATP, ATP-sensitive potassium channel; KorAHF, Korean Acute Heart Failure Registry; LDL, Low-density lipoprotein; LDL-C, low-density lipoprotein cholesterol; LIFE, Losartan Intervention for Endpoint Reduction in Hypertension Study; MHC, major histocompatibility complex; MI, myocardial infarction; Na+/K+-ATPase, sodium/potassium adenosine-triphosphatase; NAFLD, nonalcoholic fatty liver disease; NEFA, non-esterified fatty acid; NF-κB, nuclear factor κB; NGT, normal glucose tolerance; NIDD, non-insulin dependent diabetes; NKT cells, natural killer t cells; NO, nitric oxide; NPH, neutral protamine hagedorn; O₂, oxygen; OAD, oral antidiabetic drugs; OHA, oral hypoglycemic agents; PA, physical activity; PAD, peripheral artery disease; PCOS, polycystic ovary syndrome; PGU, peripheral glucose uptake; PPAR, peroxisome proliferator-activated receptor; RAAS, renin angiotensin aldosterone system; RCTs, randomized controlled trials; ROS, reactive oxygen species; REGARDS, the REasons for Geographic and Racial Differences in Stroke; SBP, systolic blood pressure; SGLT-2, sodium-glucose co-transporter-2; TORPEDO, The Treatment of Cardiovascular Risk in Primary care using Electronic Decision Support; T1DM, type 1 diabetes mellitus; T2DM, type 2 diabetes mellitus; TG, triglycerides; TNF-α, tumor necrosis factor-α; TyG index, triglyceride–glucose index; TZD, thiazolidinediones; UKPDS, United Kingdom Prospective Diabetes Study; VLDL, very low-density lipoprotein; WC, waist circumference; WHO, World Health Organization.
